# Dental Management of Tatton–Brown–Rahman Syndrome: A Case Report

**DOI:** 10.1155/crid/1991572

**Published:** 2026-05-21

**Authors:** Raed Rafat Ghulman, Dalia Abdulkhaliq Huwaykim, Dalia Shamseldin Ali Mohamed, Heba Adel Mohammedalhassan, Omar Abd El Sadek El Meligy

**Affiliations:** ^1^ Dental Department, Prince Mohammed Bin Abdulaziz Hospital, Ministry of National Guard Health Affairs, Al-Madinah Al-Munawwarah, Saudi Arabia, ngha.med.sa; ^2^ Pediatric Dentistry and Dental Public Health Department, Faculty of Dentistry, Alexandria University, Alexandria, Egypt, alexu.edu.eg

**Keywords:** dental anomalies, DNMT3A, general anesthesia, pediatric dentistry, special health care needs, Tatton–Brown–Rahman syndrome

## Abstract

Tatton–Brown–Rahman syndrome (TBRS) is a rare autosomal dominant overgrowth disorder caused by pathogenic variants in the DNMT3A gene and characterized by intellectual disability, behavioral abnormalities, craniofacial dysmorphism, and systemic comorbidities. Dental manifestations and management strategies in affected individuals remain poorly documented. This report is aimed at describing the dental findings and multidisciplinary management of a pediatric patient with TBRS, highlighting comprehensive dental rehabilitation under general anesthesia (GA). An 11–year–old Saudi female diagnosed with TBRS presented for dental clearance prior to planned transcatheter atrial septal defect (ASD) device closure. The patient exhibited intellectual disability, autism spectrum disorder, severe communication difficulties, and definitely negative behavior, making routine dental care impossible. Clinical and radiographic examinations revealed multiple carious teeth, congenitally missing maxillary incisors, ectopic tooth eruption, abnormal root morphology, and short clinical crowns. Comprehensive dental rehabilitation, including restorative treatment, extraction, and preventive care, was successfully performed under GA following multidisciplinary medical consultation. Dental rehabilitation under GA can be safely and effectively performed in children with TBRS when careful preoperative assessment and interdisciplinary coordination are ensured.

## 1. Introduction

Tatton–Brown–Rahman syndrome (TBRS) is a rare overgrowth and intellectual disability syndrome caused by heterozygous pathogenic variants in the DNMT3A gene, first described by Tatton–Brown and colleagues in 2014 [1, 2]. DNMT3A plays a key role in DNA methylation and epigenetic regulation, and pathogenic variants are believed to disrupt normal regulation of genes involved in growth and neurodevelopment, although the precise molecular mechanisms underlying the clinical phenotype remain incompletely understood [3]. The estimated prevalence of TBRS is less than 1 per 1,000,000 individuals, making it an exceptionally rare genetic condition [3].

TBRS is typically inherited in an autosomal dominant manner, with most reported cases arising from de novo mutations [4]. Rarely, familial transmission has been described, conferring a 50% risk of inheritance to offspring of affected individuals [4]. Clinically, TBRS is characterized by prenatal and postnatal overgrowth, increased height and/or head circumference (≥ 2 standard deviations above the mean), obesity or increased body weight, and intellectual disability ranging from mild to severe.[3, 4] Additional features commonly include hypotonia, joint hypermobility, kyphoscoliosis, seizures, and a wide spectrum of behavioral and psychiatric disturbances, such as autism spectrum disorder and obsessive–compulsive behaviors [3].

Individuals with TBRS often exhibit subtle but recognizable craniofacial dysmorphism, including a round face with coarse features, thick horizontal low–set eyebrows, narrow palpebral fissures, and prominent maxillary central incisors, with the facial gestalt becoming more apparent during adolescence [4]. Systemic involvement is common, with cardiac anomalies reported in up to 74% of patients, febrile seizures in approximately 22%, and an increased risk of hematologic malignancies, particularly acute myeloid leukemia, documented in isolated cases [4, 5]. Less well–defined associations with aortic root dilatation and other solid tumors have also been suggested [4].

Despite increasing recognition of TBRS, information regarding its oral and dental manifestations remains limited. Only a small number of case reports have described dental findings and management challenges in affected individuals [6]. Children with TBRS frequently present with severe behavioral difficulties, communication impairment, and limited cooperation, which complicate conventional dental care and often necessitate advanced behavior guidance techniques or treatment under general anesthesia (GA) [6]. GA is considered an appropriate and recommended modality for dental management in patients exhibiting definitely negative behavior when comprehensive treatment is required.

The present case report describes the dental findings and comprehensive dental management under GA of a child with TBRS who required dental clearance prior to planned cardiac intervention. This report highlights previously underreported dental anomalies and emphasizes the importance of multidisciplinary coordination in achieving safe and effective full–mouth dental rehabilitation in medically and behaviorally complex pediatric patients.

## 2. Case Presentation

An 11–year–old Saudi female was referred to the pediatric dentistry clinic at Prince Mohammed Bin Abdulaziz Hospital, Al–Madinah Al–Munawwarah, Saudi Arabia, for dental evaluation prior to planned transcatheter atrial septal defect (ASD) device occlusion surgery.

### 2.1. Clinical Timeline and Management Overview

To improve clarity, the clinical course is summarized chronologically as follows: The patient initially presented for dental clearance prior to planned cardiac intervention. This was followed by comprehensive medical and genetic evaluation confirming TBRS and associated comorbidities. Due to severe behavioral limitations, detailed dental and radiographic assessment was performed, revealing multiple anomalies and treatment needs. A multidisciplinary consultation involving pediatric cardiology, anesthesiology, and pediatrics was conducted to assess fitness for GA. Comprehensive dental rehabilitation under GA was subsequently carried out, followed by postoperative monitoring and discharge. Finally, prosthetic rehabilitation was initiated to address missing anterior teeth and improve function and esthetics.

The patient had a confirmed diagnosis of TBRS based on exome sequencing, identifying a pathogenic DNMT3A variant. Her medical history was significant for global developmental delay (predominantly cognitive), autism spectrum disorder, speech disorder, hypotonia, macrocephaly, and mild behavioral disturbances. Pediatric neurology assessment confirmed global developmental delay with cognitive delay more pronounced than motor delay, autism spectrum disorder, speech and language impairment, macrocephaly, mild hypotonia, and features consistent with Asperger′s syndrome. Brain magnetic resonance imaging demonstrated periventricular leukomalacia with a small ependymal cyst in the left lateral ventricle.

### 2.2. Diagnostic Reasoning and Differential Diagnosis

Although the patient presented with clinical features suggestive of an overgrowth syndrome, several differential diagnoses were considered, including Sotos syndrome, Weaver syndrome, and other genetic conditions associated with intellectual disability, macrocephaly, and dysmorphic features. Sotos syndrome was considered due to the presence of overgrowth and developmental delay; however, the absence of characteristic facial gestalt and lack of NSD1 mutation made this diagnosis less likely. Weaver syndrome was also considered given overlapping features such as accelerated growth and craniofacial abnormalities, but typical features such as a broad forehead and camptodactyly were not prominent. Other syndromic causes of intellectual disability with behavioral disturbances were considered; however, the combination of clinical findings and confirmatory exome sequencing identifying a pathogenic DNMT3A variant established the diagnosis of TBRS. Therefore, alternative diagnoses were effectively ruled out based on genetic and phenotypic correlation.

The patient exhibited dysmorphic features, including a prominent forehead, high hairline, long nose with prominent nasal bridge, cupped ears, tapering fingers, flat feet, and clumsy gait. Developmental milestones were delayed; independent sitting was achieved at 14 months and walking at 2 years of age. Speech was characterized by poor articulation and limited comprehension of abstract concepts.

Cardiac evaluation revealed congenital heart disease with multifenestrated secundum ASD measuring approximately 20 mm in total, associated with right atrial and ventricular dilatation. Echocardiography demonstrated two large secundum ASDs in close proximity, with normal biventricular systolic function and no evidence of pulmonary hypertension. Electrocardiography showed sinus rhythm, normal axis, QTc of 421 ms, heart rate of 96 bpm, and incomplete right bundle branch block, with no atrioventricular block, arrhythmia, or ischemic changes. The patient was asymptomatic from a cardiac perspective, with no signs of right heart failure. Pediatric cardiology classified the patient as standard risk for GA at the time of dental treatment, with no indication for infective endocarditis prophylaxis. This decision was consistent with current cardiology guidelines, which recommend prophylaxis only for patients at high risk of adverse outcomes from infective endocarditis [7].

**Figure 1:**
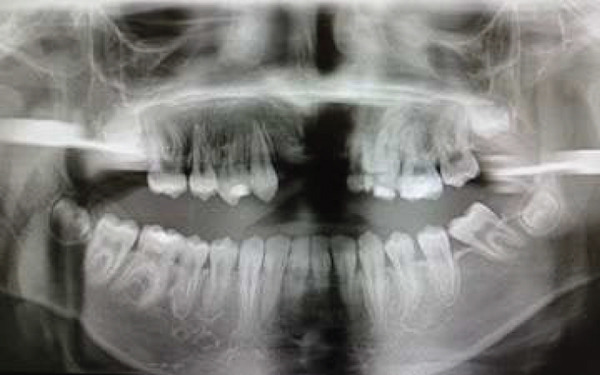
Preoperative panoramic radiograph showing multiple dental anomalies in a child with Tatton–Brown–Rahman syndrome.

**Figure 2:**
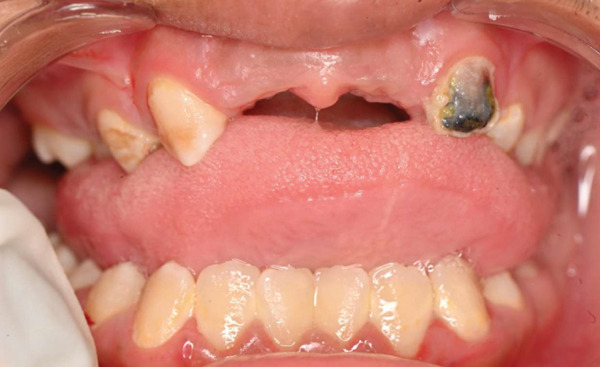
Preoperative intraoral frontal view showing poor oral hygiene, plaque accumulation, and carious maxillary primary teeth.

**Figure 3:**
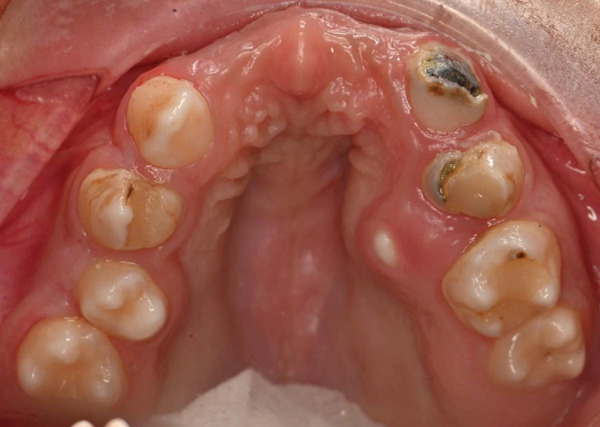
Preoperative maxillary occlusal view demonstrating multiple carious lesions and congenitally missing maxillary incisors.

On dental evaluation, the patient presented with extremely uncooperative behavior, classified as Frankl Behavior Rating Scale I (definitely negative), precluding clinical examination under conventional behavioral guidance [8]. She was unable to follow instructions, engaged in irrelevant speech, and demonstrated continuous movement within the operatory, rendering conventional dental examination impossible. Panoramic radiographic examination revealed congenital absence of maxillary permanent incisors (#11, #12, #21, and #22), proximal caries and root resorption of primary Tooth #64, palatally ectopic eruption of Tooth #24, split root morphology in Tooth #34, enlarged pulp chamber with suspected internal resorption in Tooth #35, radiopaque material within the root canal of Tooth #45, and short clinical crowns of permanent first molars (Figure 1). Poor oral hygiene and plaque accumulation were evident, particularly in the mandibular anterior region (Figure 2). Maxillary occlusal examination demonstrated multiple carious lesions and congenitally missing maxillary incisors (Figure 3).

Based on the above clinical, radiographic, and behavioral findings, a definitive treatment plan was formulated.

Following multidisciplinary consultation with pediatric cardiology, anesthesiology, and pediatrics, comprehensive dental treatment under GA was planned to eliminate all potential sources of oral infection prior to cardiac intervention. Preanesthetic evaluation revealed no previous anesthetic complications, no known drug allergies, and no current medications. The patient adhered to standard preoperative fasting guidelines, with a fasting period of 6 h prior to induction.

Preoperative laboratory investigations, including complete blood count, coagulation profile, and basic metabolic panel, were within acceptable limits for surgery and revealed no clinically significant abnormalities. Hemoglobin, platelet count, coagulation parameters, serum electrolytes, renal function, and blood glucose levels were deemed appropriate for proceeding with GA.

Under GA, the following procedures were performed: Class I composite restorations on Teeth #16, #26, #36, #46, #14, #23, and #65 using a light‐cured resin composite (Filtek Z350 XT, 3 M ESPE, United States), followed by finishing and polishing to achieve appropriate anatomical contour and surface smoothness; extraction of nonrestorable Tooth #64. Tooth #23 was identified as a permanent maxillary left canine based on its anatomical position and radiographic features, including root length, morphology, and stage of development. Although a retained primary canine was considered, particularly given the presence of hypodontia and coronal destruction, the absence of typical primary tooth features, such as a shorter, more divergent root and larger pulp chamber, supported its classification as a permanent rather than a primary tooth.

**Figure 4:**
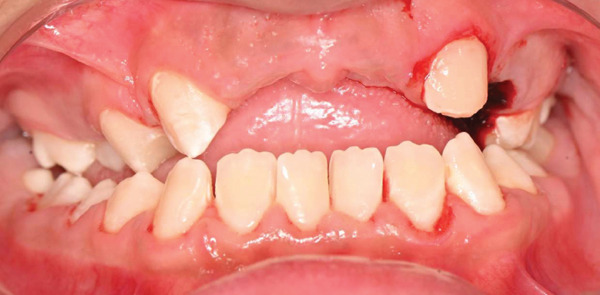
Immediate postoperative frontal view showing extraction of nonrestorable Tooth #64 and nonsurgical scaling and selective enamel disking of mandibular anterior teeth to improve plaque control.

**Figure 5:**
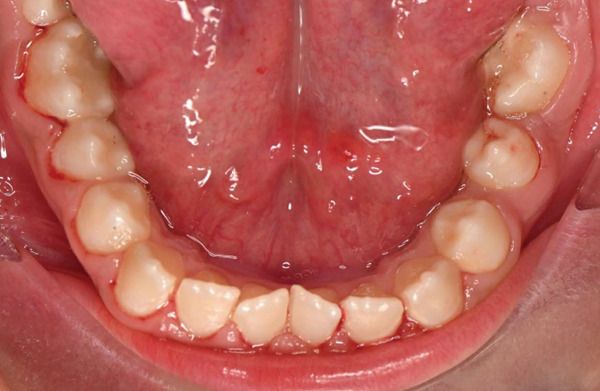
Immediate postoperative mandibular occlusal view showing scaling and selective enamel disking of mandibular anterior teeth to improve plaque control.

**Figure 6:**
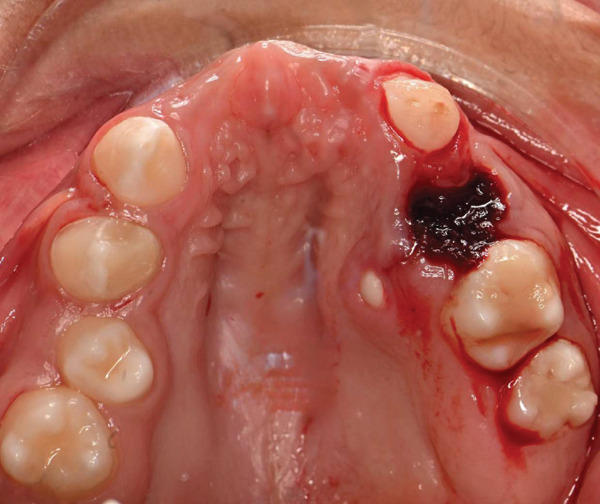
Immediate postoperative maxillary occlusal view showing surgical site management with hemostatic material.

Nonsurgical scaling and selective enamel disking of the mandibular anterior teeth were performed to reduce plaque‐retentive areas and facilitate oral hygiene, particularly given the patient′s limited cooperation and poor plaque control. The enamel reduction was conservative and confined to enamel, with minimal interproximal reduction to avoid compromising tooth structure. Surgical site management was performed using absorbable hemostatic material. Local anesthesia was administered using 2% lidocaine with 1:80,000 epinephrine (Figures 4, 5, and 6).

The procedure was completed uneventfully. The patient recovered well and was transferred to the pediatric intensive care unit for postoperative monitoring. She was discharged the following day in stable condition. Postoperative instructions, oral hygiene education, dietary counseling, and scheduled follow‐up appointments were provided to the caregiver.

**Figure 7:**
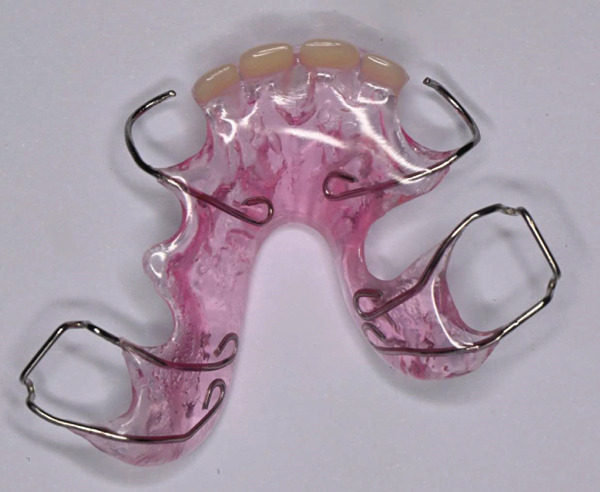
Maxillary removable prosthesis showing acrylic base, artificial teeth, and retentive clasps designed to replace congenitally missing maxillary incisors.

**Figure 8:**
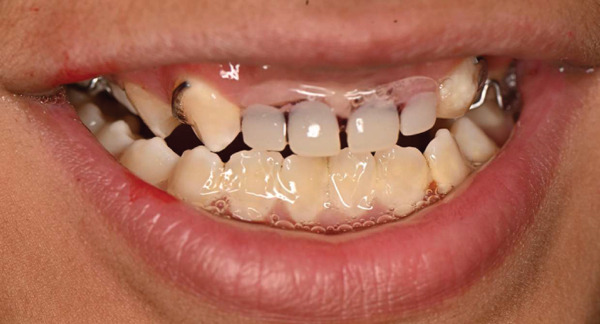
Intraoral frontal view demonstrating placement of the removable maxillary prosthesis, showing adaptation, retention, and occlusal relationship.

**Figure 9:**
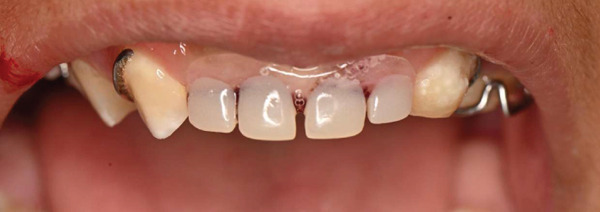
Intraoral close‐up view showing the esthetic and functional outcome following prosthetic rehabilitation in a child with Tatton–Brown–Rahman syndrome.

Following recovery from GA, prosthetic rehabilitation was initiated to address the congenitally missing maxillary incisors. Due to the patient′s limited cooperation, a conventional impression was obtained using alginate under careful behavioral management and with caregiver assistance. A removable maxillary prosthesis was subsequently fabricated with an acrylic base, artificial teeth, and retentive clasps. During delivery, particular attention was given to ensuring proper fit, retention, and occlusal harmony, with adjustments made as needed. The caregiver was instructed on appliance insertion, removal, and maintenance to support compliance and oral hygiene (Figures 7, 8, and 9). At 3‐month follow‐up, the patient demonstrated satisfactory adaptation to the removable prosthesis, with good retention and improved esthetics. Oral hygiene had improved compared with baseline, and the restorations remained clinically stable with no evidence of secondary caries or failure.

## 3. Discussion

TBRS presents significant challenges in dental management owing to the combination of intellectual disability, behavioral disturbances, and associated systemic conditions. Most published descriptions of oral and craniofacial features in TBRS emphasize the presence of prominent maxillary central incisors as a characteristic finding [4]. In contrast, the present case demonstrated congenitally missing maxillary central and lateral incisors, representing an unusual dental presentation that appears to be rarely reported in the existing literature. Existing reports of dental findings in TBRS have primarily focused on prominent maxillary incisors, eruption disturbances, and behavioral challenges affecting dental care, with limited characterization of root morphology or tooth agenesis patterns. These observations broaden the spectrum of dental anomalies associated with TBRS and underscore the importance of comprehensive dental assessment in affected individuals [1].

In addition to tooth agenesis, several previously unreported radiographic findings were identified, including split root morphology in Tooth #34, an enlarged pulp chamber and root canal in Tooth #35 suggestive of internal resorption, and unexplained intracanal radiopacity in Tooth #45.

To the best of our knowledge, such findings have been infrequently described in the literature, and their association with TBRS remains poorly characterized. These observations may reflect underlying developmental disturbances related to DNMT3A mutations, which are known to affect cellular differentiation and growth, [1] and warrant further investigation to determine whether these findings represent coincidental anomalies or part of a broader dental phenotype in TBRS.

Eruption disturbances have been inconsistently reported in TBRS. In the present case, palatally ectopic eruption of Tooth #24 was observed, a finding consistent with the report by Paz–Alegría et al. [6], who described palatal ectopic eruption of the maxillary lateral incisor (#12) in a child with TBRS. Such similarities suggest that altered eruption patterns may be an underrecognized feature of the syndrome.

Behavioral management represents a major challenge in children with TBRS due to intellectual disability, autism spectrum disorder, hyperactivity, and limited cooperation [2]. To date, only one published report has described dental management in a child with TBRS using conventional behavioral guidance techniques [6]. In contrast, the severity of behavioral difficulties in the present case, combined with the urgent need to eliminate potential sources of oral infection prior to planned transcatheter ASD device occlusion, necessitated comprehensive dental rehabilitation under GA.

GA remains an essential and well–established modality for delivering safe and effective dental care in children with special health care needs who cannot tolerate treatment under local anesthesia or conscious sedation [9, 10]. In medically complex patients such as those with TBRS and associated congenital heart disease, careful preoperative planning and multidisciplinary coordination are critical. In this case, close collaboration between pediatric dentistry, anesthesiology, pediatric cardiology, and pediatric neurology was fundamental in ensuring patient safety and achieving successful treatment outcomes.

The dental treatment plan in this case focused on eliminating infection, restoring function, and facilitating long–term oral hygiene. Given the lack of syndrome–specific dental management guidelines for TBRS, treatment decisions were individualized. Although Paz–Alegría et al. [6] reported the use of multiple composite restorations and a modified Sander appliance, appliance–based or removable prosthetic options were considered unsuitable in the present case because of the patient′s poor compliance and behavioral challenges. Interproximal reduction of the lower anterior teeth was performed to reduce plaque retention and improve cleansability, prioritizing oral hygiene and disease prevention over complex restorative or orthodontic interventions.

Preventive dental care and caregiver education remain the cornerstone of long–term management in children with TBRS [11]. The poor oral hygiene index observed in this patient can be attributed to limited manual dexterity, cognitive impairment, hyperactivity, and difficulty maintaining a consistent daily oral hygiene routine. Caregivers were therefore extensively counseled on the importance of supervised tooth brushing, the use of child–friendly and adaptive oral hygiene aids, dietary sugar reduction, and the need for regular dental follow–up visits. These measures are essential to minimize the risk of recurrent dental disease and reduce the likelihood of future interventions under GA, which carry increased medical and financial burdens.

### 3.1. Clinical Implications and Key Takeaways

This case highlights several important clinical considerations for TBRS. First, dental anomalies in TBRS may extend beyond the commonly reported features, emphasizing the need for thorough clinical and radiographic assessment. Second, significant behavioral challenges frequently necessitate advanced behavior guidance techniques, including treatment under GA, to ensure safe and effective care. Third, the presence of associated systemic conditions, particularly congenital heart disease, underscores the importance of careful preoperative assessment and multidisciplinary coordination. Early recognition of oral health needs, combined with preventive strategies and caregiver education, is essential to reduce disease burden and minimize the need for repeated interventions under GA.

## 4. Conclusions

TBRS is a rare genetic condition with distinctive systemic and craniofacial features. This case highlights previously underreported dental anomalies and demonstrates that comprehensive dental rehabilitation under GA can be safely achieved through meticulous planning and multidisciplinary collaboration. Early preventive care and continuous caregiver involvement are essential to improving oral health outcomes and quality of life in affected children.

## Author Contributions

All authors contributed to the conception, clinical management, data collection, manuscript drafting, and final approval of the submitted version.

## Funding

No funding was received for this manuscript.

## Consent

Written informed consent was obtained from the patient′s parents/legal guardians for publication of this case report and accompanying images.

## Conflicts of Interest

The authors declare no conflicts of interest.

## Data Availability

The data that support the findings of this study are available from the corresponding author upon reasonable request.
